# Characterization of microbiome diversity unveils substantial microbial variation in mangrove soil sediments from coastal regions of Malaysia

**DOI:** 10.1099/acmi.0.000902.v3

**Published:** 2025-06-18

**Authors:** Prashantha Hebbar, Oh Bi Han, Ng Xin Yan, Dominic Kay, Kwa Yee Chu, James Sy-Keen Woon, Pang Kok Lun, Shama Prasada Kabekkodu, Alevoor S. Bharath Prasad, Bharathi Prakash, Nadine Nograles, Mahibub Mahamadsa Kanakal, Michaela Goodson, Shubhada Nagaraja, Roshan Mascarenhas

**Affiliations:** 1 Mbiomics LLC, 16192 Coastal Highway, Lewes, Delaware 19958, USA; 2 Manipal Centre for Biotherapeutics Research, Manipal Academy of Higher Education, Manipal, Karnataka, 576104, India; 3 Newcastle University Medicine Malaysia (NUMed), 1, Jalan Sarjana 1, Kota Ilmu, 79200 Iskandar Puteri, Johor, Malaysia; 4 Faculty of Medical Sciences, Newcastle University, Newcastle upon Tyne, NE1 7RU, UK; 5 Department of Cell and Molecular Biology, Manipal School of Life Sciences, Manipal Academy of Higher Education, Manipal, 576104, Karnataka, India; 6 Department of Microbiology, University College, Mangalore, Karnataka, 575001, India; 7 Faculty of Pharmacy, Quest International University, Ipoh, 30250, Malaysia

**Keywords:** 16S sequencing, coastal Malaysia, ecosystem, mangrove microbiome, microbial diversity, soil bacteria

## Abstract

The mangrove ecosystems are of great ecological importance found in tropical and subtropical coasts, including Malaysia. The microbial communities in the mangrove sediments play an indispensable role in maintaining homeostasis and supporting biodiversity. However, mangroves are facing various threats due to increasing anthropogenic activities. Thus, it is important to monitor the microbial community to improve our understanding of anthropogenic pressure on reshaping these ecosystems. This study examines the microbial community diversity in mangrove sediments of southern peninsular Malaysia. High-throughput MinION sequencing of the 16S rRNA gene was performed to compare the soil microbiome diversity in 35 samples from 8 different mangroves representing Sungai Sedili Kecil and Sungai Sedili Besar that flow into the South China Sea; Sungai Pulai, Sungai Melayu, Sungai Danga, Sungai Skudai and Sungai Johor that join the Straits of Johor; and Pulau Kukup from the Straits of Malacca. The metagenomic classification performed with 16S rRNA showed 2,573 taxa comprising 32 phyla. Total abundance analysis showed *Pseudomonadota* (67–69%), *Bacteroidota* (6–8%), *Bacillota* (5–8%), *Campylobacterota* (4–5%), *Acidobacteriota* (3–4%), *Planctomycetota* (2–4%) and *Actinomycetota* (1–2%) as the relatively common phyla. Alpha diversity indices revealed significantly higher richness in samples from mangroves of the South China Sea. Further, the ‘Shannon’ index showed a significant difference in diversity between Sungai Melayu and Sungai Pulai. Higher abundance of *Burkholderiaceae*, *Bacillaceae* and *Enterobacteriaceae* suggests a difference in the microbial community structure. This study stands as the first comprehensive analysis of microbial communities for future monitoring and conservation in these mangroves.

## Data Summary

The authors confirm that all supporting data and protocols have been provided within the article. The genomic raw reads files from this study are publicly available at the Sequence Read Archive of the National Center for Biotechnology Information under the study BioProject ID: PRJNA1139970. The following are the distinct identification numbers for sequence records.

SRS24660329, SRS22117015, SRS22117016, SRS22117017, SRS22117018, SRS22117019, SRS22117020, SRS22117021, SRS22117022, SRS22117023, SRS22117024, SRS22117025, SRS22117026, SRS22117027, SRS22117028, SRS22117029, SRS22117030, SRS22117031, SRS22117032, SRS22117033, SRS22117034, SRS22117035, SRS22117036, SRS22117037, SRS22117038, SRS22117039, SRS22117040, SRS22117041, SRS22117042, SRS22117043, SRS22117044, SRS22117045, SRS22117046, SRS22117047, SRS22117048.

## Introduction

Mangrove forests are unique coastal ecosystems at the interface between terrestrial and marine environments, supporting a diverse array of plant and animal life, resulting in a unique biodiversity [1,2]. Mangrove soil sediments provide a niche for microbial communities that are key drivers of nutrient cycling and organic matter decomposition and possess a large capacity to store carbon, which gets them the name ‘blue carbon reservoirs’ [3]. The mangrove ecosystem, comprising 10–15% of global carbon storage capacity, predominantly stores up to two-thirds of its organic carbon in soils [4,5]. Therefore, soil microbiomes are critical for the optimum operation of biogeochemical cycles due to microbes that are particularly involved in carbon, nitrogen, phosphorus and sulphur cycles. These capabilities of the mangrove ecosystem also play an important role in mitigating climate change [6,7]. Soil microbiomes are responsible for overall ecosystem resilience, and thus, there is a need for their conservation [8].

The coastal regions of Malaysia harbour extensive mangrove forests that are known for their remarkable biodiversity and ecological importance but continue to show about 0.41% decline per year [9]. Southeast Asia is recognized as a global hotspot of mangrove loss due to fragmentation resulting from the conversion of forests to aquaculture and agriculture. Malaysia is reported to be one of the top countries to show aerial loss and high rates of fragmentation in mangrove forests [10]. In peninsular Malaysia, Johor is the southernmost state with the second largest mangrove by area with 32,301 ha spread over several distinct microhabitats. This also includes three out of seven Ramsar sites (wetlands of international importance), namely Pulau Kukup, Tanjung Piai and Sungai Pulai [11]. Numerous estuaries and the unique geographical location of Johor favour mangroves on all three sides, facing the Straits of Malacca on the west, the Straits of Johor in the south and the South China Sea in the east. However, despite their ecological significance, the microbiome diversity of mangrove soils in southern peninsular Malaysia remains relatively understudied.

Understanding the composition and dynamics of microbial communities is crucial for comprehending the functioning and resilience of mangrove ecosystems, particularly in the face of environmental challenges [12]. Though there are numerous conservation and rehabilitation efforts in Malaysia such as gazetting mangroves as permanent reserve forests and mangrove replanting projects, there is still continued damage and an increasing number of threats including fragmentation of mangroves, heavy metals, petroleum hydrocarbons, microplastic pollution, palm plantations, aquaculture, industries and residential developments [13,16]. Therefore, it is likely that there will be a difference in the structure of the mangrove microbiome influenced by anthropogenic pressure, and knowing the indicator strains is useful for monitoring the environmental impact over time [17].

Metagenomics enables the identification of microbial taxa at high resolutions, providing insights into their diversity, abundance and potential functional roles [18]. A metagenomic study on mangrove microbiome composition revealed mainly bacteria (96.24%), archaea (2.78%) and eukaryotes, viruses and unclassified organisms (0.98%) [19]. Studies have shown that mangrove microbiomes have a unique microbial profile compared to terrestrial and ocean biomes due to different microbial community assembly mechanisms [20,21]. In addition, there is a wide difference in microbial diversity between different mangroves mainly due to differences in pH and type of plant species [22,23]. Metagenomics has also shown the variation in microbial profile between healthy and damaged mangrove, which makes it important to explore and document mangrove microbiome profiles from different geographical locations before they are further damaged [24]. The current study provides insights into the microbiome diversity in mangrove sediments from the southern coastal regions of peninsular Malaysia.

## Methods

### Site characterization and sample collection

**Fig. 1.**
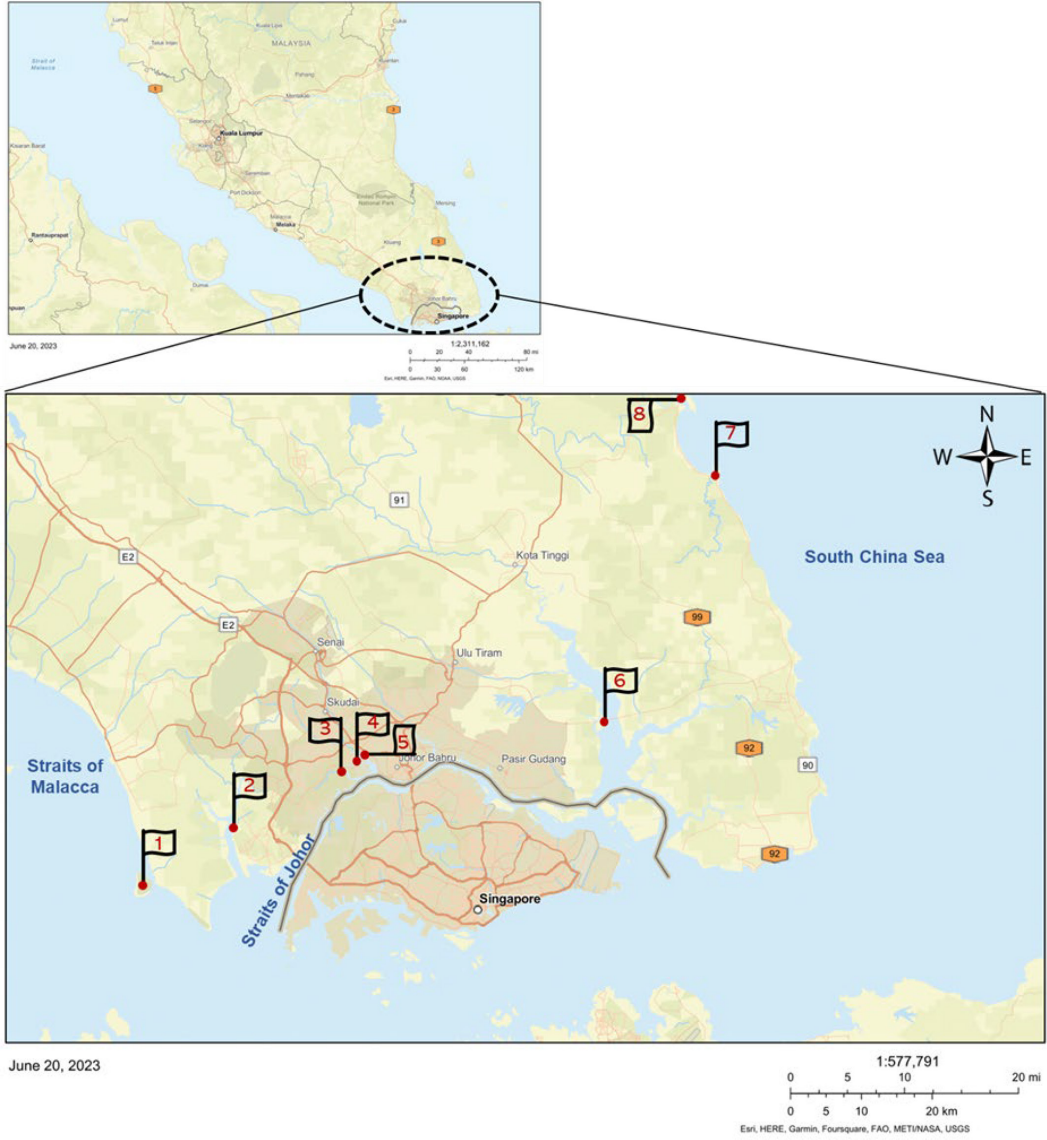
Geographical location of the sampling regions. Mangrove soil sediments were collected from eight different mangroves in southern peninsular Malaysia. Numbers indicate sampling area: 1, Pulau Kukup (sm.pk); 2, Sungai Pulai (sj.*sp*); 3, Sungai Melayu (sj.sm); 4, Sungai Danga (sj.sd); 5, Sungai Skudai (sj.ss); 6, Sungai Johor (sj.sj); 7, Sungai Sedili Kecil (scs.ssk); and 8, Sungai Sedili Besar (scs.ssb). Sampling region 1 is facing the sm, 2–6 the sj and 7–8 the scs. Map image generated using ArcGIS. Basemap sources: Esri, HERE, Garmin, FAO, NOAA, USGS.

Soil sediments were collected between June and September 2022 during the dry weather conditions from eight mangroves located in geographic regions along the South China Sea (scs), Straits of Johor (sj) and Straits of Malacca (sm) in southern peninsular Malaysia as shown in Fig. 1. Sampling locations were chosen based on the anthropogenic risks and the accessibility of sampling points within mangrove ecosystems. For consistency, all samples were collected during flood current when the incoming tide moves towards the shore. Triplicate topsoil samples were collected for each site as described previously [25]. Water physical parameters such as pH, dissolved oxygen (DO) and conductivity were measured *in situ* using a HACH HQ40D portable metre (Hach Company, CO, USA), and the temperature was recorded using the handheld multiparameter metre (YSI, USA). Permission for sample collection at Kukup Island was obtained from Johor National Parks, Malaysia.

A total of 35 samples were collected, of which 6 were from Sungai Sedili Besar (scs.ssb) (*sungai* means ‘river’ in Malay) and 3 from Sungai Sedili Kecil (scs.ssk), together 9 representative samples from 2 locations of scs. Further, 3 samples from Sungai Pulai (sj.*sp*), 8 from Sungai Melayu (sj.sm), 2 from Sungai Danga (sj.sd), 2 from Sungai Skudai (sj.ss) and 5 samples from Sungai Johor (sj.sj), together 20 representative samples from 5 locations of sj, and 6 representative samples from Pulau Kukup (sm.pk) location from sm were collected. Each sample is derived by pooling triplicate soil samples per site. The difference in the number of samples collected from each study site was primarily due to variations in mangrove coverage, site complexity and associated anthropogenic pressures. A minimum of six samples were collected per region to ensure baseline representation. However, sites such as sj.sm, which encompass a larger mangrove area with multiple converging rivulets and greater exposure to anthropogenic activities, had more sampling points. In contrast, sj.sd and sj.ss are connected and have limited mangrove areas, resulting in fewer sampling points. Descriptions of sampling points and characteristics are given in Table S1, available in the online Supplementary Material.

### DNA extraction and MinION sequencing

Genomic DNA was extracted using a FastDNA SPIN Kit for Soil and a FastPrep-24 Homogeniser (MP Biomedicals, CA, USA). The sequencing library was prepared using a MinION 16S rRNA kit from Oxford Nanopore Technologies (ONT, Oxford, UK) as per the manufacturer’s instructions. For each sample, 20 ng of DNA was subjected to PCR amplification with a 16S Barcoding kit (SQK-16S024) using LongAmp 2X Master Mix (New England Biolabs, Herts, UK). The amplified library was purified using Agencourt AMPure XP (Beckman Coulter, CA, USA), pooled and loaded onto the MinION sequencing flow cell (R9.4.1, FLO-MIN106). The average reads were obtained using a MinION sequencing device (MinION Mk1B) supported with MinKNOW software for data acquisition. The output data was extracted in FAST5 file format for each barcode and was used for analysis.

### Sequence quality control and processing

Albacore software ver. 2.3.4 (Oxford Nanopore Technologies) with the Guppy algorithm was used for base calling the MinION™ sequencing data (FAST5 files) to generate pass reads in FASTQ format. Multiple FASTQ sequences per run were concatenated to form a single FASTQ file per run. Quality control of the MinION data was carried out using FastQC to ensure read quality was within the expected bounds. The trim adapter and barcode sequences from the ends of reads, and also to split reads with internal adapters (or chimaeras), into separate reads, the Porechop tool was used. For quality filtering of sequence reads, the fastp tool was used [26]. Reads with a minimum average read quality score greater than 9 were retained as recommended [27]. Further, minimum sequence length ≥1,000 bp and maximum sequence length ≤2,000 bp were kept for retaining 1,300–1,950 bp sequences for the V1-V9 region. Owing to differences in algorithms in identifying chimaeras, we further validated the resulting fastq files with *de novo* chimaera detection using the vsearch v2.27.0 tool. Read distributions of each sample were compared using NanoComp and plotted using ggplot2 [28].

### Taxonomy classification

We employed the RDP Classifier (version 2.14) [29], a Naïve Bayesian classifier, for taxonomic classification. This classifier integrates newly described taxa and recent revisions in prokaryotic nomenclature by utilizing the updated RDP taxonomy training set no. 19, which was released in 2023. The update comprises 24,642 sequences covering 19,074 species and 3,903 genera. The taxonomy assignment confidence cutoff was set to 0.5, and the assignment count for each taxon was obtained in the hierarchical tab-delimited format. This data was exported to the R environment for further quality filtering and statistical analyses using the Phyloseq package. We identified potential outlier samples by determining taxa abundance in each sample and then filtering abundance <Q1-1.5×IQR or abundance >Q3-1.5×IQR across samples. We removed features with ambiguous phylum annotation, such as either ‘uncharacterized’ or ‘chloroplast’ being classified as *Cyanobacteria*. We performed rarefaction for the challenges using the rare_even_depth() function available within the Phyloseq package [30]. Rarefaction normalizes the number of sequences across samples, allowing for fairer comparison of taxonomic assignments and diversity metrics. The ggplot2 was used for the visualization of microbiome data.

### Diversity estimation and statistical analysis of diversity across regions

The alpha diversity of samples was measured by ‘Observed’, ‘Chao1’ and ‘Shannon’ diversity indices. Additionally, beta diversity was determined and visualized using principal coordinate analysis (PCoA) plots based on unweighted UniFrac distances. To test whether the observed diversity differs significantly between regions, we performed a non-parametric test, the Wilcoxon rank-sum test (Mann–Whitney) on ‘Observed’, ‘Chao1’ and ‘Shannon’ indices across regions. Similarly, to evaluate individual phylum differences between regions. Further, a cross-location diversity comparison was also performed among regions of sj and scs.

## Results

### Characteristics of mangrove soil samples

Samples from representative sites of different mangrove locations showed dark brown soil. Surface water properties such as temperature (28.8–30.9 ℃), pH (6.18–6.92), DO (4.77–7.21 mg l^−1^) and electrical conductivity (28.7–35.5 mS/cm) were relatively similar except for sj.sd and sj.ss, which recorded low DO (Table S2). Anthropogenic threats were medium to high for mangroves on the Straits of Johor due to rapid urbanization, land reclamation, domestic, industrial and agricultural waste, while it remains low to moderate for the Straits of Malacca mangroves at Kukup Island and the South China Sea, primarily due to lower levels of urbanization in these areas.

### Sequencing and quality control

Our MinION sequencing experiments yielded 7,881,578 total number of reads with an average of 218,932 reads per sample and 1,422.8 bases average read length. Regarding the Q score, all the reads showed *Q*>7 in all the samples, of which 73% of the reads showed *Q*>10 and 17% of the reads showed *Q*>12. After retaining quality sequences with parameters based on mean quality score, minimum and maximum sequence length, 7,379,232 reads with an average of 210,835 reads per sample were retained. This exercise enhanced average read length to 1,440.87 bases and the proportion of *Q*>10 to 73.37% and *Q*>12 to 17.31%. Regarding individual regions, we observed scs, sj and sm comprising 224,107, 218,187 and 178,036 reads. The distribution of mean *Q* scores and mean read lengths for all samples is shown in Fig. S1A, B. Furthermore, the quality-filtered representative samples were employed to classify all input reads by aligning or matching the information content in reads to the RDP reference database using taxonomic sequence classifiers. The frequencies are shown in Table S3.

### 16S rRNA taxonomy classification

Across 35 samples, we identified 3,587 taxa. We excluded taxa associated with ‘Eukaryota’ and ‘unclassified_Root’, as well as those classified as chloroplasts within the *Cyanobacteria* order, leaving 3,497 taxa spanning 54 phyla. Quality filtering based on taxa abundance across samples revealed no outliers. Subsequent rarefaction removed an additional 936 taxa, resulting in 2,573 taxa spanning 32 phyla available for downstream diversity analysis. Of the phyla, we found *Acidobacteriota*, *Actinomycetota*, *Bacillota*, *Bacteroidota*, *Campylobacterota*, *Planctomycetota* and *Pseudomonadota* were present greater than 1% of the relative abundances, while *Armatimonadota*, *Atribacterota*, *Calditrichota*, *Chlamydiota*, *Chloroflexota*, *Chlorobiota*, *Cyanobacteriota*, *Deferribacterota*, *Deinococcota*, *Elusimicrobiota*, *Fibrobacterota*, *Fusobacteriota*, *Gemmatimonadota*, *Kiritimatiellota*, *Lentisphaerota*, *Mycoplasmatota*, *Nitrospinota*, *Nitrospirota*, *Rhodothermota*, *Spirochaetota*, *Synergistota*, *Thermodesulfobacteriota*, *Thermomicrobiota*, Thermoproteota and *Verrucomicrobiota* were present in less than 1% of the relative abundance across 35 samples. The mean prevalence and total abundance of phyla with a relative occurrence greater than 0.2% across all samples are shown in Table 1.

**Table 1.** Mean prevalence and total abundance of phyla with a relative occurrence greater than 0.2% across all samples

Phylum	Mean occurrence of each phylum across all the samples	Total abundance across samples	Relative occurrence of each phylum across all the samples (in %)
*Nitrospinota*	27.75	2,267	0.2
*Verrucomicrobiota*	12.57	2,266	0.2
*Calditrichota*	29	2,310	0.2
*Chlorobiota*	26.14	3,690	0.3
*Chloroflexota*	13.57	6,711	0.5
*Cyanobacteriota*	6.54	7,159	0.5
*Spirochaetota*	19.88	7,561	0.6
*Gemmatimonadota*	28	7,638	0.6
*Kiritimatiellota*	29.08	9,358	0.7
*Nitrospirota*	27.9	9,419	0.7
*Actinomycetota*	10.63	13,231	1.0
*Planctomycetota*	20.8	35,511	2.7
*Acidobacteriota*	17.61	47,315	3.6
*Campylobacterota*	25.96	61,730	4.7
*Bacteroidota*	11.47	99,519	7.6
*Bacillota*	10.58	101,315	7.7
*Pseudomonadota*	13.65	894,707	67.9

### Bacterial community structure across scs, sj and sm

The phylum‐level composition of the microbiomes across three regions is shown in Fig. S2. Of the 32 phyla, only 4 phyla, including *Pseudomonadota* (67–69%), *Bacteroidota* (6–8%), *Bacillota* (5–8%) and *Campylobacterota* (4–5%), showed relative abundance >4% in all three regions. However, only the proportional composition of *Bacillota* (scs, 8.6%; sj, 8.1%; and sm, 5.2%) showed a noticeable difference in total mean abundance. Further location-wise analysis among eight different mangroves showed variability in mean phylum distribution (Fig. S3). A striking difference was seen across five mangrove locations of the sj region. *Pseudomonadota* in different locations ranged from 62.1 to 72.6%, with the lowest in sj.sj (62.1%) and highest in sj.sd (72.6%). *Bacillota* showed high in sj.ss (11.56%), sj.sm (10.66%) and low in sm.pk (5.2%), sj.*sp* (4.52%) and sj.sj (4.89%). *Campylobacterota* was high in sj.ss (11.14%) and sj.sd (6.26%) and low in sj.sj (2.8%), sj.*sp* (3.29) and sm.pk (4.06). *Acidobacteriota* was low in sj.ss (1.1%), sj.sm (2.19%) and sj.sd (2.36%) compared to sj.sj (5.57%) and sj.*sp* (5.36%). *Planctomycetota* high in sj.sj (4.86%), sm.pk (4.04%) and sj.*sp* (3.16%) and low in sj.ss (0.87%), sj.sd (1.32%) and sj.sm (1.6%). *Actinomycetota* was found high in sm.pk (1.45%) and sj.*sp* (1.43%) compared to sj.sd (0.42%) and sj.ss (0.58%).

**Fig. 2.**
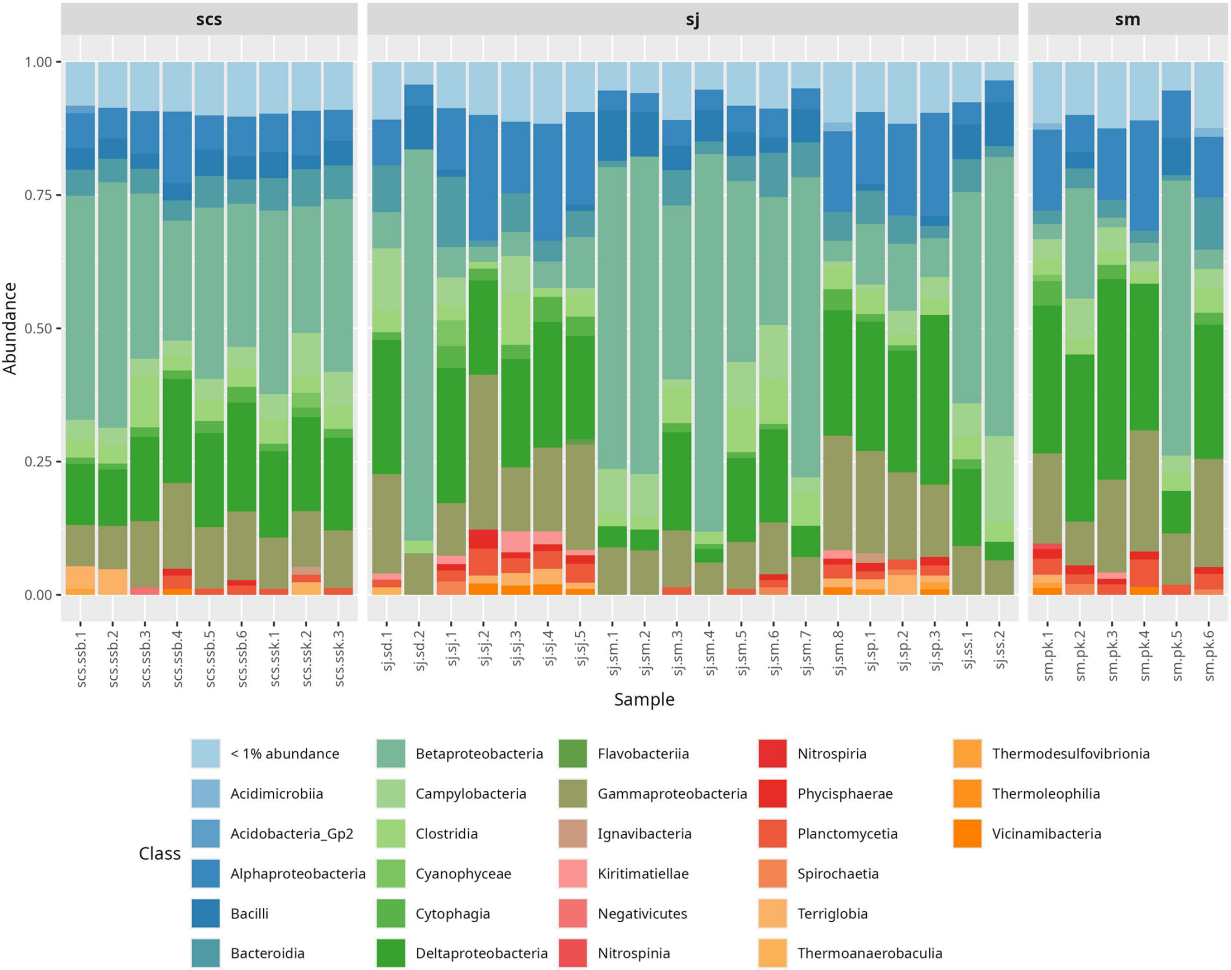
Relative abundance of bacterial classes in different samples. Bar plot showing the relative abundance of the core microbiome at the class level: scs, sj and sm.

At the class level, the most abundant taxa across all three regions included *Betaproteobacteria* (27%), *Deltaproteobacteria* (17.48%), *Gammaproteobacteria* (12.81%), *Alphaproteobacteria* (9.77%), *Campylobacteria* (5.33%), *Bacteroidia* (4.88%), *Clostridia* (3.87%) and *Bacilli* (3.64%), although their relative proportions varied among sites (Fig. 2). *Betaproteobacteria* was the dominant class in all scs locations. Within sj sites, however, the pattern differed: sj.sm showed a composition similar to scs, while sj.sj (5.52%) and sj.*sp* (10.39%) had notably lower levels of *Betaproteobacteria* and higher proportions of *Alphaproteobacteria* (17.56% in sj.sj and 16.69% in sj.*sp*). In both these sites, *Deltaproteobacteria* emerged as the most abundant class, ranging between 21 and 26%.

**Fig. 3.**
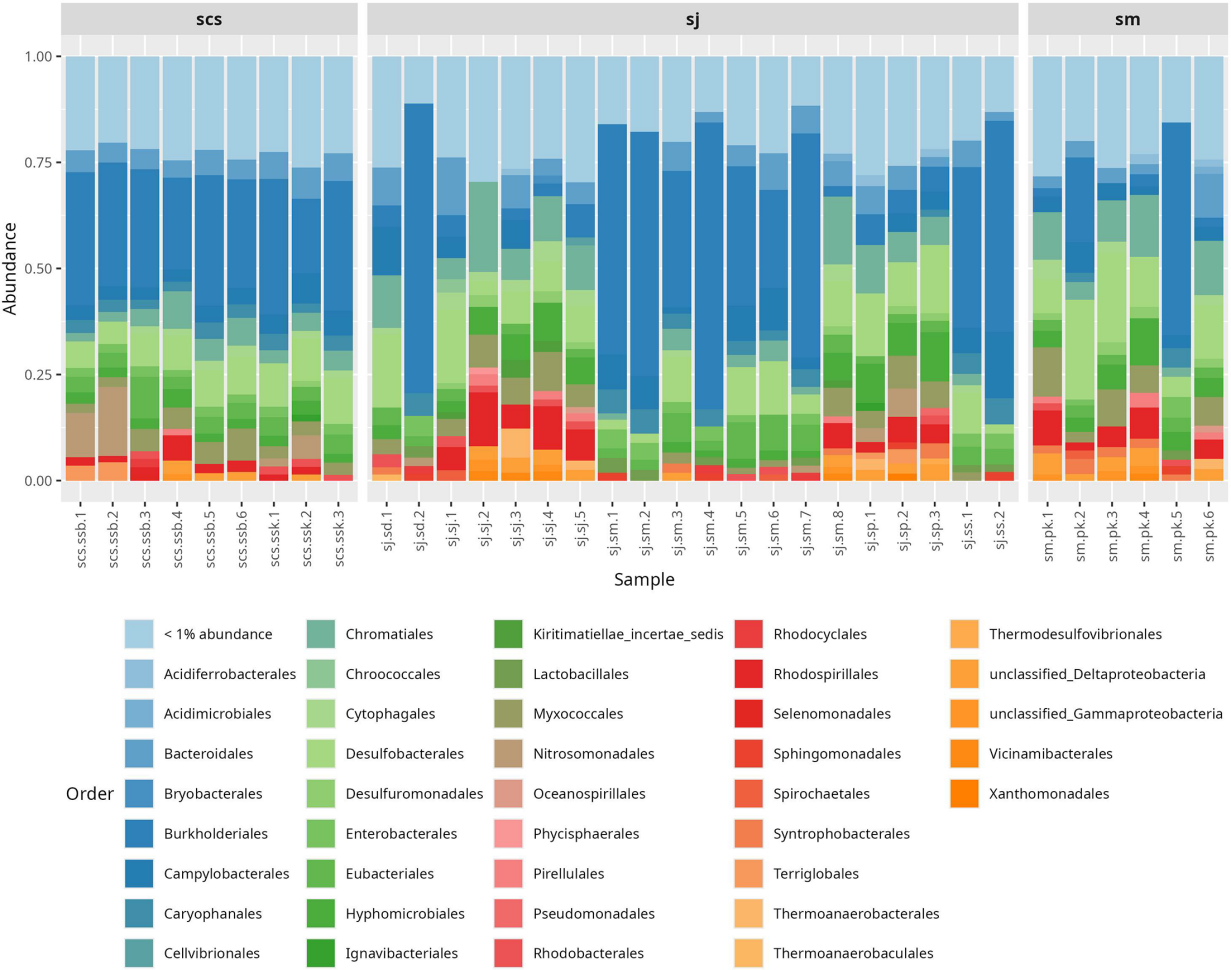
Relative abundance of bacterial orders in different samples. Bar plot showing the relative abundance of the core microbiome at the order level: scs, sj and sm.

At the order level, the dominant taxa included *Burkholderiales* (18.15%), *Desulfobacterales* (6.73%), *Chromatiales* (4.39%), *Campylobacterales* (3.72%), *Bacteroidales* (3.63%) and *Hyphomicrobiales* (2.81%) (Fig. 3). Interestingly, *Burkholderiales* was significantly more abundant in sites located closer to urban areas and exposed to domestic wastewater, such as sj.sd (27.33%), sj.ss (32.38%) and sj.sm (29.51%), compared to more remote locations like sj.sj (2.85%), sj.*sp* (4.42%) and sm.pk (9.46%). A similar trend was observed for *Campylobacterales*, with higher relative abundance in sj.ss (7.99%), sj.sd (4.46%) and sj.sm (3.68%) than in sj.sj (1.85%), sj.*sp* (2.32%) and sm.pk (2.73%).

**Fig. 4.**
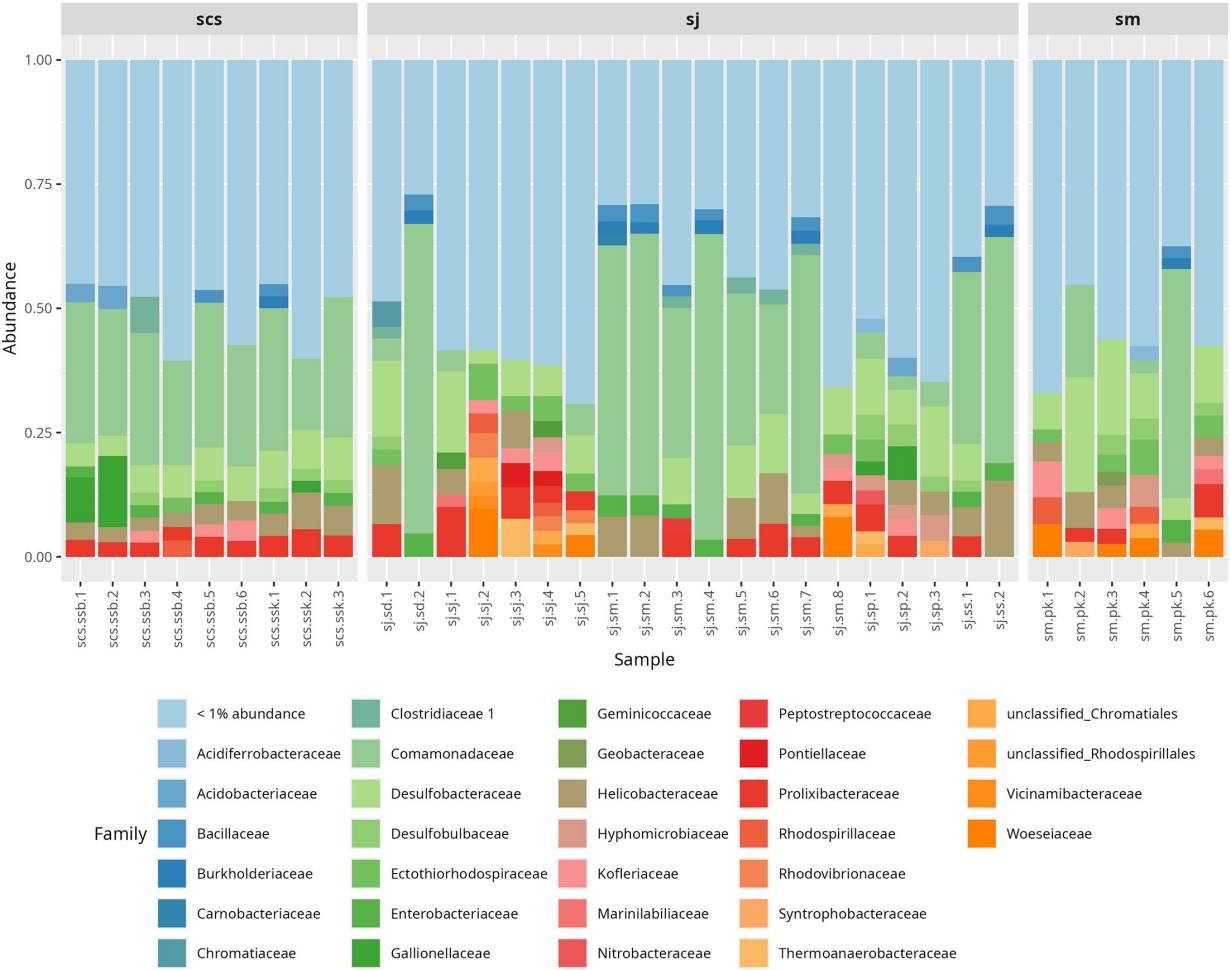
Relative abundance of bacterial families in different samples. Bar plot showing the relative abundance of the core microbiome at the family level: scs, sj and sm.

At the family level, *Comamonadaceae*, *Desulfobacteraceae*, *Helicobacteraceae*, *Prolixibacteraceae*, *Enterobacteriaceae*, *Burkholderiaceae* and *Bacillaceae* were among the predominant bacterial families identified (Fig. 4). Notably, *Comamonadaceae* showed higher relative abundance in sj.ss (19.11%), sj.sd (16.31%) and sj.sm (17.52%) compared to sj.sj (1.27%), sj.*sp* (1.86%) and sm.pk (5.5%). Similarly, *Bacillaceae* was more abundant in sj.ss (1.63%), sj.sd (0.92%) and sj.sm (1.05%) than in sj.sj (0.24%), sj.*sp* (0.41%) and sm.pk (0.36%). *Enterobacteriaceae* followed the same trend, with higher levels in sj.ss (1.59%), sj.sm (1.16%) and sj.sd (1.23%) compared to sj.*sp* (0.16%), sm.pk (0.51%) and sj.sj (0.09%). *Burkholderiaceae* also showed greater abundance in sj.ss (1.07%), sj.sd (0.73%) and sj.sm (0.84%) than in sj.sj (0.07%), sj.*sp* (0.08%) and sm.pk (0.26%). Interestingly, *Helicobacteraceae* was notably higher in sj.ss (5.1%) compared to sj.*sp* (1.55%), sm.pk (1.73%) and sj.sj (1.18%) and select samples of sj.sm and sj.sd.

In contrast, families such as *Desulfobacteraceae*, *Desulfobulbaceae* and *Ectothiorhodospiraceae* were more abundant in sj.*sp* and sm.pk than in sj.ss, sj.sm and sj.sd. For instance, *Desulfobacteraceae* was found at higher levels in sj.*sp* (4.63%) and sm.pk (5.27%) compared to sj.ss (2.18%) and sj.sm (2.85%). *Desulfobulbaceae* was also elevated in sj.*sp* (1.77%) and sm.pk (1.0%) relative to sj.ss (0.58%), sj.sm (0.32%) and sj.sd (0.58%). *Ectothiorhodospiraceae* showed greater abundance in sj.*sp* (1.29%), sm.pk (1.35%) and sj.sj (1.64%) compared to sj.ss (0.1%) and sj.sm (0.43%).

Among the less abundant families, *Geobacteraceae* exhibited higher levels in sj.*sp* (0.47%), sm.pk (0.58%) and sj.sj (0.64%) than in sj.ss (0.17%), sj.sm (0.18%) and sj.sd (0.18%). Similarly, *Vicinamibacteraceae* was more prevalent in sj.*sp* (0.43%), sm.pk (0.43%) and sj.sj (0.75%) compared to sj.ss (0.04%), sj.sm (0.2%) and sj.sd (0.24%).

At the genus level, the most abundant genera included *Atlantibacter*, *Defluviicoccus*, *Desulfatiglans*, *Desulfatitalea*, *Ferruginivarius*, *Gp23*, *Kofleria*, *Limibacillus*, *Mangrovibacillus*, *Nitrospira*, *Pelagibius*, *Puteibacter*, *Sideroxyarcus*, *Sulfuriflexus*, *Sulfurovum*, *Thermoanaerobaculum*, *Thioalbus*, *Thiobacter*, *Vicinamibacter* and *Woeseia*.

### Bacterial richness, evenness, and beta diversity across samples

**Fig. 5.**
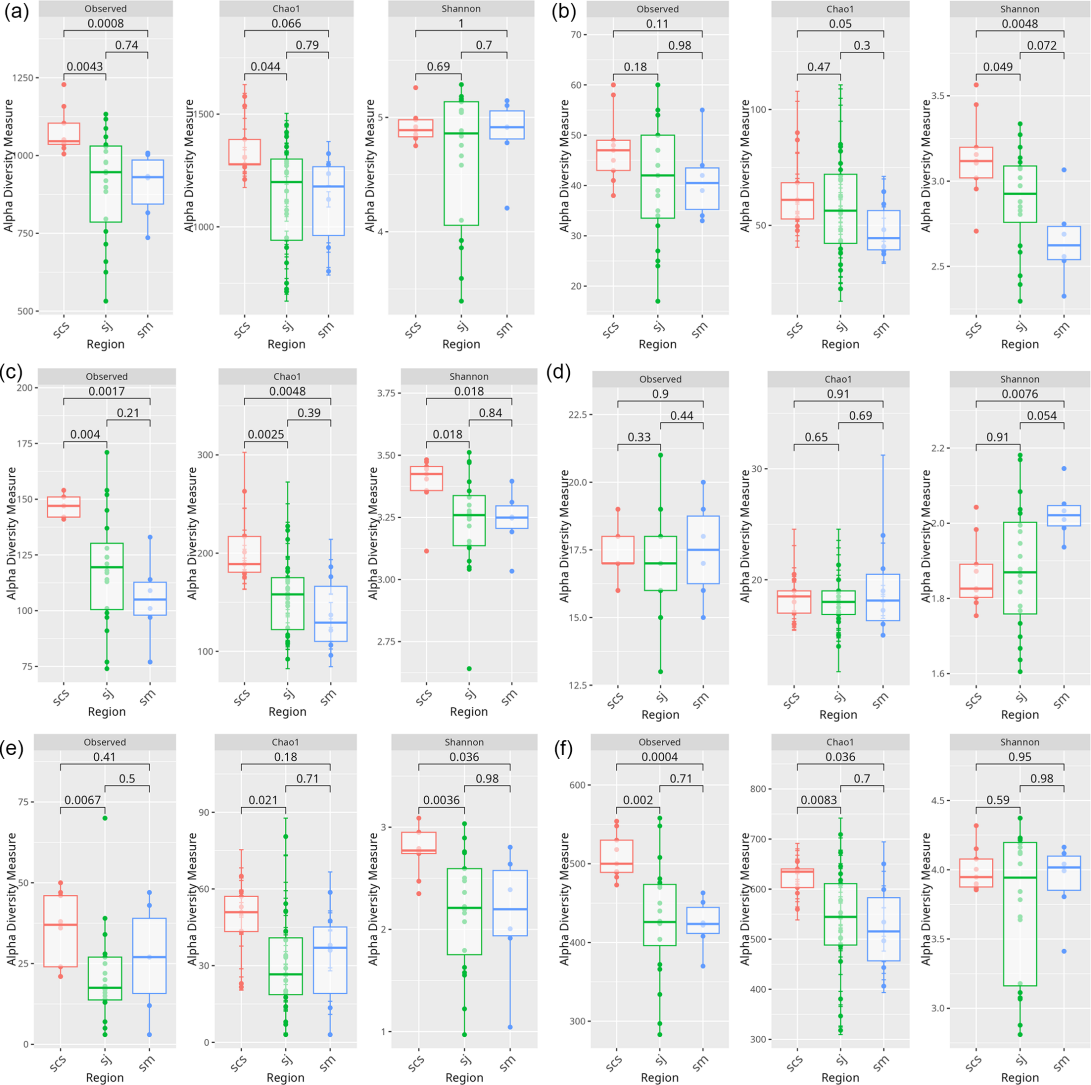
Delineating alpha diversity across three regions of mangrove soil samples with Observed, Chao1 and Shannon index measures, where Chao1 is an abundance-based estimator of species richness and Shannon index is an estimator of species richness and species evenness. Box plots showing regional cross-comparison of alpha diversity indices (**a**) overall comparison showing significantly higher richness in scs compared to sj and sm. (**b**) *Actinomycetota*, (**c**) *Bacillota*, (**d**) *Campylobacterota*, (**e**) *Cyanobacteriota*, (**f**) *Pseudomonadota*. Statistical significance was defined as *P* < 0.05.

Interestingly, the sj region, which comprised a greater number of sequences compared to sm, displayed the lowest ‘Shannon’ index, indicating that this microbial community is the least diverse. The richness indices such as ‘Observed’ showed high values for scs with significant difference compared to sj (*P*=0.0043), sm (*P*=0.008) and ‘Chao1’ for scs vs. sj (*P*=0.044) (Fig. 5a). Among the abundant phyla, the alpha diversity measures such as ‘Observed’ and ‘Chao1’ showed significant difference in richness across three regions for *Actinomycetota*, *Bacillota*, *Campylobacterota*, *Cyanobacteriota* and *Pseudomonadota*. *Actinomycetota* showed a difference with ‘Chao1’ for scs vs. sm (*P*=0.05), ‘Shannon’ for scs higher compared to sj (*P*=0.049) and sm (*P*=0.0048) (Fig. 5b). For *Bacillota*, ‘Observed’ (scs vs. sj, *P*=0.004, scs vs. sm, *P*=0.0017) and ‘Chao1’ (scs vs. sj, *P*=0.0025, scs vs. sm, *P*=0.0048) showed significant differences in richness. ‘Shannon’ showed significant differences in evenness between sj and sm (*P*=0.018) (Fig. 5c). As regards *Campylobacterota*, we did not find any differences in richness across regions with both ‘Observed’ and ‘Chao1’, whereas ‘Shannon’ showed higher evenness in sm compared to scs (*P*=0.0076) (Fig. 5d). In *Cyanobacteriota*, ‘Observed’ showed difference between scs vs. sj (*P*=0.006), ‘Chao1’ scs vs. sj (*P*=0.021), ‘Shannon’ sj (*P*=0.003) and sm (*P*=0.036) (Fig. 5e). With *Pseudomonadota*, ‘Observed’ showed a significant difference between scs vs. sm (*P*=0.0004) and scs vs. sj (*P*=0.002), and ‘Chao1’ showed between scs vs. sj (*P*=0.008), scs vs. sm (*P*=0.036) in richness, whereas ‘Shannon’ showed no significant difference in evenness across the three regions (Fig. 5f).

Across locations of sj, alpha diversity indices by ‘Observed’ showed sj.*sp* significantly diverse than sj.sm (*P*=0.033). Further, ‘Chao1’ also showed sj.*sp* significantly diverse than sj.sm (*P*=0.033) and sj.sj diverse than sj.sm (*P*=0.042). ‘Shannon’ index measure showed sj.*sp* significantly higher in richness and evenness compared to sj.sm (*P*=0.017) and sj.sj higher compared to sj.sm (*P*=0.0061) (Fig. S4).

**Fig. 6.**
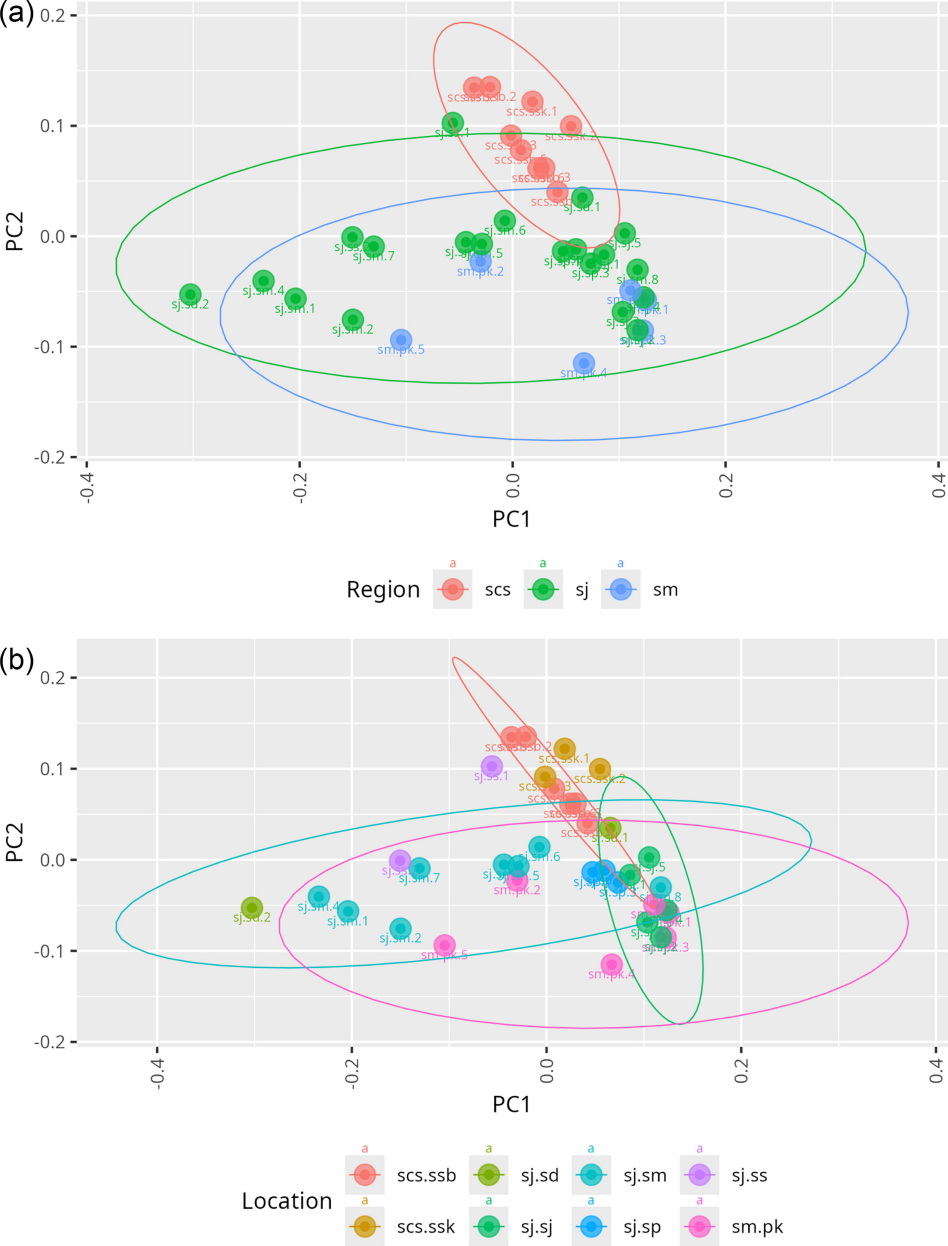
PCoA plots of beta diversity based on unweighted UniFrac distances. (**a**) Region-wise comparison of beta diversity in mangrove sediment microbiomes across three regions: scs, sj and sm. (**b**) Location-wise comparison of beta diversity among specific sampling sites: scs.ssb, scs.ssk, sj.sd, sj.sj, sj.sm, sj.*sp*, sj.ss and sm.pk. Each dot represents an individual mangrove sediment sample. Samples are enclosed within ellipses indicating groupings by region (**a**) or location (**b**) to visualize community structure differences.

Further beta diversity analysis highlights the variation in microbial community composition across three regions (Fig. 6a). Distinct clustering patterns were evident in the scs and sm regions, indicating pronounced beta diversity between these two mangrove soils. In contrast, the sj region displayed a broader range of beta diversity and showed partial overlap with both scs and sm, suggesting that its microbial communities are more admixed. This pattern points to the potential influence of geographical and anthropogenic factors on microbial composition. Likewise, Fig. 6b presents the beta diversity across various locations within the scs, sj and sm regions.

## Discussion

In Malaysia, mangroves are threatened by large-scale anthropogenic disturbances such as coastal development, logging, farming, urbanization and aquaculture resulting in declining ecosystem health, eventually causing degradation of this valuable environmental asset [10,16]. In this study, we present the structure of the microbiome in mangrove soil and offer insights into the microbial community within mangroves located in some of the fastest-developing regions of Malaysia.

*Pseudomonadota* (previously *Proteobacteria*), known for their essential role in nitrogen fixation within mangrove ecosystems, were found to be consistently abundant across various regions, including the scs, the sm and the sj, aligning with the global core mangrove microbiome composition. This pattern is supported by studies from diverse locations such as the Beibu Gulf, the Sundarbans in India and mangrove forests in Brazil, where *Pseudomonadota* comprise more than half of the microbial abundance regardless of sampling season or restoration status [18,21, 31,33]. Additionally, other studies have shown that certain physicochemical factors, such as varying salinity levels, do not significantly alter the abundance of *Pseudomonadota*, which remains relatively stable at around 55.33% [19]. In contrast, research in Panama Bay revealed significantly reduced proportions (<30%) of *Pseudomonadota* in urban mangroves exposed to pollution, highlighting the influence of anthropogenic activity on microbial community structure [24].

Despite rapid urbanization, the overall abundance of *Pseudomonadota* appears relatively resilient in various mangrove locations of the sj – possibly due to the early stages of pollution exposure and the recent onset of mangrove degradation. Nevertheless, differences in the relative proportions of different *Pseudomonadota* classes have been observed. Anthropogenic factors have been shown to alter the relative abundance of different classes within *Pseudomonadota*. While studies in China, India and Brazil have reported *Gammaproteobacteria* and *Deltaproteobacteria* as the predominant classes, our study identifies *Betaproteobacteria* as the predominant class among phylum *Pseudomonadota*, followed by *Deltaproteobacteria* and *Gammaproteobacteria* [18,20, 34]. This difference may be linked to increasing urbanization and pollution, as also observed in highly anthropized mangroves of Brazil and India [20,35, 36]. Notably, the order *Burkholderiales* (within *Betaproteobacteria*) showed higher abundance in sj.sm, sj.ss and sj.sd, all subject to increasing domestic wastewater pollution and urban development. Given their ability to degrade hydrocarbons, pesticides and organic pollutants, *Burkholderiales* may serve as bioindicators of environmental stress [20,36]. Thus, monitoring these changes can provide insights into the health of the mangrove ecosystem.

Furthermore, the presence of higher *Campylobacterota* (previously *Epsilonproteobacteria*), particularly of order *Campylobacterales*, in all three regions, with the dominant genus *Sulfurovum*, indicates their role in detoxification. *Sulfurovum* bacteria are chemolithoautotrophic and are mainly involved in the transformation of sulphur compounds, contributing to nutrient cycling and overall ecosystem functioning. The increased presence of *Campylobacterota* could be an indicator of mangrove ecosystems actively managing environmental stresses as part of their restoration [24].

Studies have indicated *Planctomycetota* as decontaminating agents in mangroves by degrading petroleum-derived hydrocarbon pollutants as well as being involved in methane and sulphur metabolism [17,37]. Our study reveals higher levels of *Planctomycetota* in mangroves closer to higher boat traffic, such as sj.sj, sm.pk and sj.*sp*, and low in urbanized locations, such as sj.ss, sj.sd and sj.sm, differing from previous findings that associate low *Planctomycetota* levels with pristine and regenerated mangroves [31,32].

The area surrounding sm.pk has previously been documented to exhibit elevated levels of total petroleum hydrocarbons and increased oil and grease (O and G) pollution, largely attributed to boat traffic and aquaculture activities in adjacent waters, although recent studies suggest these levels are declining [13,38]. In contrast, the sj region has experienced pollution from ammoniacal nitrogen (NH₃-N) and phosphate (PO₄) runoff, largely stemming from palm oil plantations, as well as polycyclic aromatic hydrocarbon contamination from marine traffic in the sj [39,40].

The higher relative abundance of sulphate-reducing bacteria, such as *Desulfobacteraceae*, *Desulfobulbaceae* and *Ectothiorhodospiraceae*, observed in oil-affected mangrove areas such as sm.pk, sj.*sp* and sj.sj suggests that these bacterial families are strongly associated with anaerobic, hydrocarbon-rich sediments [22]. Their presence in these environments highlights their role in sulphur cycling and petroleum hydrocarbon degradation and may serve as potential bioindicators of sediment stability and health in less sewage-impacted, yet organically enriched, sediments.

In contrast, mangrove areas exposed to domestic wastewater and sewage, including sj.ss, sj.sd and sj.sm – showed lower levels of sulfate-reducing bacteria but higher abundance of facultative and opportunistic taxa such as *Comamonadaceae*, *Enterobacteriaceae*, *Bacillaceae*, *Burkholderiaceae* and *Helicobacteraceae*. These families, particularly *Enterobacteriaceae*, are enriched in polluted environments rich in organic waste, heavy metals or urban effluents, making them effective indicators of anthropogenic pressure [41,42].

Furthermore, *Geobacteraceae*, an anaerobic metal-reducing family, and *Vicinamibacteraceae*, a group within the phylum *Acidobacteriota*, typically found in organic-rich and relatively undisturbed sediments, were more abundant in sm.pk and sj.*sp*, while their presence was markedly reduced in sj.ss, sj.sd and sj.sm. These differences suggest that both families may be sensitive to environmental degradation, especially in settings where oxidation, toxic substances or heavy anthropogenic inputs disrupt anaerobic microbial communities. Their distribution supports their role as indicators of ecological stability and sediment health in mangrove ecosystems.

These microbial patterns correspond with observed variations in richness and diversity across the sj region, particularly highlighting ecological and anthropogenic contrasts between sj.sj and sj.sm, as well as sj.*sp* and sj.sm. The beta diversity analysis revealed significant differences in microbial communities among the three regions, indicating that regional environmental variability likely plays a significant role in shaping microbial community structures. These differences may be attributed to variations in organic content or human-related impacts across the regions. Geographically, the sj could be a crossover point for microbiome seen in the scs and the sm. Hence, it has a more admixed pattern and is rich in microbial communities. Within the sj, sj.*sp*, a designated RAMSAR site, displayed the highest diversity, while sj.sm, adjacent to the rapidly urbanizing Iskandar region, was the least diverse [11,16].

One limitation of this study is the use of 16S rRNA amplicon sequencing, which can pose challenges in accurate assignment of genus and may bias the detection towards more abundant taxa when studying complex microbial ecosystems with a high degree of diversity.

Altogether, our study suggests the intricate relationship between microbial communities in mangroves and anthropogenic threats. Differences in microbial compositions, particularly increased betaproteobacteria and *Burkholderiales*, suggest the impact of anthropogenic influence on mangrove microbiomes. Further experiments on microbial interaction and measuring the levels of different types of chemical pollutants may help to understand factors influencing the restructuring of these mangrove microbiomes. These findings emphasize the need for concerted efforts in mangrove conservation to mitigate human-induced alterations in these delicate ecosystems.

## Conclusion

Our study provides an important foundational understanding of the mangrove microbiome structure in Malaysia. We consider this as the first study to report the microbial community structure in these mangroves exposed to rapid urbanization. In conclusion, this study provides valuable insights into the dynamics of microbial communities in mangroves exposed to different anthropogenic threats. The observed differences among various classes of *Pseudomonadota*, higher *Campylobacterota* and the families *Burkholderiaceae*, *Bacillaceae* and *Enterobacteriaceae* serve as indicators of microbiome restructuring in response to pollution and urbanization. Monitoring these changes not only enhances our understanding of mangrove ecosystem health but also provides a basis for developing conservation and restoration strategies to mitigate the impact of anthropogenic stressors on these vital ecosystems.

## Supplementary material

10.1099/acmi.0.000902.v3 Uncited Supplementary Material 1.

10.1099/acmi.0.000902.v3 Uncited Supplementary Material 2.

## Abbreviations

DO: dissolved oxygen
PCoA: principal coordinate analysis
scs: South China Sea
scs.ssb: Sungai Sedili Besar
scs.ssk: Sungai Sedili Kecil
sj: Straits of Johor
sj.sd: Sungai Danga
sj.sj: Sungai Johor
sj.sm: Sungai Melayu
sj.sp: Sungai Pulai
sj.ss: Sungai Skudai
sm: Straits of Malacca
sm.pk: Pulai Kukup

## References

[1] Carugati L, Gatto B, Rastelli E, Lo Martire M, Coral C (2018). Impact of mangrove forests degradation on biodiversity and ecosystem functioning. Sci Rep.

[2] Nagelkerken I, Blaber SJM, Bouillon S, Green P, Haywood M (2008). The habitat function of mangroves for terrestrial and marine fauna: a review. Aquatic Botany.

[3] Nellemann C, Corcoran E, Duarte CM, Valdés L, De Young C (2009). Blue carbon. A rapid response assessment.

[4] Alongi DM (2014). Carbon cycling and storage in mangrove forests. Annu Rev Mar Sci.

[5] Sasmito SD, Kuzyakov Y, Lubis AA, Murdiyarso D, Hutley LB (2020). Organic carbon burial and sources in soils of coastal mudflat and mangrove ecosystems. *CATENA*.

[6] Feller IC, Friess DA, Krauss KW, Lewis RR (2017). The state of the world’s mangroves in the 21st century under climate change. Hydrobiologia.

[7] Howard J, Sutton-Grier A, Herr D, Kleypas J, Landis E (2017). Clarifying the role of coastal and marine systems in climate mitigation. Frontiers in Ecol & Environ.

[8] Trevathan-Tackett SM, Sherman CDH, Huggett MJ, Campbell AH, Laverock B (2019). A horizon scan of priorities for coastal marine microbiome research. Nat Ecol Evol.

[9] Hamilton SE, Casey D (2016). Creation of a high spatio‐temporal resolution global database of continuous mangrove forest cover for the 21st century (CGMFC‐21). Global Ecol Biogeogr.

[10] Bryan-Brown DN, Connolly RM, Richards DR, Adame F, Friess DA (2020). Global trends in mangrove forest fragmentation. Sci Rep.

[11] Ramli SF, Caihong Z (2017). National mangrove restoration project in malaysia. J Environ Earth Sci.

[12] Wainwright BJ, Millar T, Bowen L, Semon L, Hickman KJE (2023). The core mangrove microbiome reveals shared taxa potentially involved in nutrient cycling and promoting host survival. Environ Microbiome.

[13] Fadzil MF, Yun PS, Razal AR, Chee PS, Suratman S (2017). Oil and grease and total petroleum hydrocarbons in the waters of ramsar gazetted mangrove area. Johor J Sustain Sci Manag.

[14] Gonsilou AP, Azman S, Isharunizam NIA, Mohamed Najib MZ, Syafiuddin A (2023). Microplastic uptake by mud creepers (Cerithidea obtusa) at Kukup, Johor. J Sustain Sci Manag.

[15] Halim NHA, Abdullah R, Kadir WR, Ajeng AA, Zawawi NZB (2022). Heavy metals distribution and fractionation in mangrove sediments linked to organic deposits vis-à-vis accumulation in *Rhizophora* spp. at Tanjung Piai, Johor, Malaysia. Appl Ecol Env Res.

[16] Kanniah K, Sheikhi A, Cracknell A, Goh H, Tan K (2015). Satellite images for monitoring mangrove cover changes in a fast growing economic region in southern Peninsular Malaysia. Remote Sens.

[17] de Araujo JE, Taketani RG, Pylro VS, Leite LR, Pereira E Silva M de C (2021). Genomic analysis reveals the potential for hydrocarbon degradation of *Rhodopirellula* sp. MGV isolated from a polluted Brazilian mangrove. Braz J Microbiol.

[18] Gong B, Cao H, Peng C, Perčulija V, Tong G (2019). High-throughput sequencing and analysis of microbial communities in the mangrove swamps along the coast of Beibu Gulf in Guangxi, China. Sci Rep.

[19] Sepúlveda-Correa A, Daza-Giraldo LV, Polanía J, Arenas NE, Muñoz-García A (2021). Genes associated with antibiotic tolerance and synthesis of antimicrobial compounds in a mangrove with contrasting salinities. Marine Pollution Bulletin.

[20] Imchen M, Kumavath R, Barh D, Azevedo V, Ghosh P (2017). Searching for signatures across microbial communities: metagenomic analysis of soil samples from mangrove and other ecosystems. Sci Rep.

[21] Zhang C-J, Pan J, Duan C-H, Wang Y-M, Liu Y (2019). Prokaryotic diversity in mangrove sediments across southeastern china fundamentally differs from that in other biomes. mSystems.

[22] Fiard M, Cuny P, Sylvi L, Hubas C, Jézéquel R (2022). Mangrove microbiota along the urban-to-rural gradient of the Cayenne estuary (French Guiana, South America): drivers and potential bioindicators. Sci Total Environ.

[23] Liu M, Huang H, Bao S, Tong Y (2019). Microbial community structure of soils in Bamenwan mangrove wetland. Sci Rep.

[24] Quintero IJ, Castillo AM, Mejía LC (2022). Diversity and taxonomy of soil bacterial communities in urban and rural mangrove forests of the Panama Bay. Microorganisms.

[25] Bhattacharyya A, Haldar A, Bhattacharyya M, Ghosh A (2019). Anthropogenic influence shapes the distribution of antibiotic resistant bacteria (ARB) in the sediment of Sundarban estuary in India. Sci Total Environ.

[26] Chen S, Zhou Y, Chen Y, Gu J (2018). fastp: an ultra-fast all-in-one FASTQ preprocessor. Bioinformatics.

[27] Nygaard AB, Tunsjø HS, Meisal R, Charnock C (2020). A preliminary study on the potential of Nanopore MinION and Illumina MiSeq 16S rRNA gene sequencing to characterize building-dust microbiomes. Sci Rep.

[28] De Coster W, D’Hert S, Schultz DT, Cruts M, Van Broeckhoven C (2018). NanoPack: visualizing and processing long-read sequencing data. Bioinformatics.

[29] Wang Q, Garrity GM, Tiedje JM, Cole JR (2007). Naive Bayesian classifier for rapid assignment of rRNA sequences into the new bacterial taxonomy. Appl Environ Microbiol.

[30] Schloss PD (2024). Rarefaction is currently the best approach to control for uneven sequencing effort in amplicon sequence analyses. mSphere.

[31] Basak P, Pramanik A, Sengupta S, Nag S, Bhattacharyya A (2016). Bacterial diversity assessment of pristine mangrove microbial community from Dhulibhashani, Sundarbans using 16S rRNA gene tag sequencing. Genom Data.

[32] Jeyanny V, Norlia B, Getha K, Nur-Nabilah A, Lee SL (2020). Bacterial communities in a newly regenerated mangrove forest of Sungai Haji Dorani mangroves in the West Coast of Selangor, Malaysia. J Trop Forest Sci.

[33] Ma X-X, Jiang Z-Y, Wu P, Wang Y-F, Cheng H (2021). Effect of mangrove restoration on sediment properties and bacterial community. Ecotoxicology.

[34] De Santana CO, Spealman P, Melo V, Gresham D, de Jesus T (2021). Large-scale differences in diversity and functional adaptations of prokaryotic communities from conserved and anthropogenically impacted mangrove sediments in a tropical estuary. PeerJ.

[35] Mughini-Gras L, van der Plaats RQJ, van der Wielen PWJJ, Bauerlein PS, de Roda Husman AM (2021). Riverine microplastic and microbial community compositions: a field study in the Netherlands. Water Res.

[36] Nogueira VLR, Rocha LL, Colares GB, Angelim AL, Normando LRO (2015). Microbiomes and potential metabolic pathways of pristine and anthropized Brazilian mangroves. Reg Stud Mar Sci.

[37] Rampadarath S, Bandhoa K, Puchooa D, Jeewon R, Bal S (2018). Metatranscriptomics analysis of mangroves habitats around Mauritius. World J Microbiol Biotechnol.

[38] Manan NMR, Yuh YS, Theng LW, Zakaria MP (2011). Distribution of petroleum hydrocarbons in aquaculture fish from selected locations in the straits of malacca, malaysia. World Appl Sci J.

[39] Keshavarzifard M, Zakaria MP, Keshavarzifard S (2019). Evaluation of polycyclic aromatic hydrocarbons contamination in the sediments of the Johor Strait, Peninsular Malaysia. Polycyclic Aromat Compd.

[40] Samsudin et al (2017). River water quality assessment using APCS-MLR and statistical process control in johor river basin, malaysia. Int J Adv Appl Sci.

[41] Ghaderpour A, Mohd Nasori KN, Chew LL, Chong VC, Thong KL (2014). Detection of multiple potentially pathogenic bacteria in Matang mangrove estuaries, Malaysia. Mar Pollut Bull.

[42] Meng S, Peng T, Pratush A, Huang T, Hu Z (2021). Interactions between heavy metals and bacteria in mangroves. Marine Pollution Bulletin.

