# Supplementary material for: Characterization of microbiome diversity unveils substantial microbial variation in mangrove soil sediments from coastal regions of Malaysia
Source: Access Microbiol. 2025 Jun 18;7(6):000902.v3. doi: 10.1099/acmi.0.000902.v3 (PMC12281800; doi:10.1099/acmi.0.000902.v3)
Supplement: Uncited Supplementary Material 1. [file acmi-7-00902-s001.pdf]

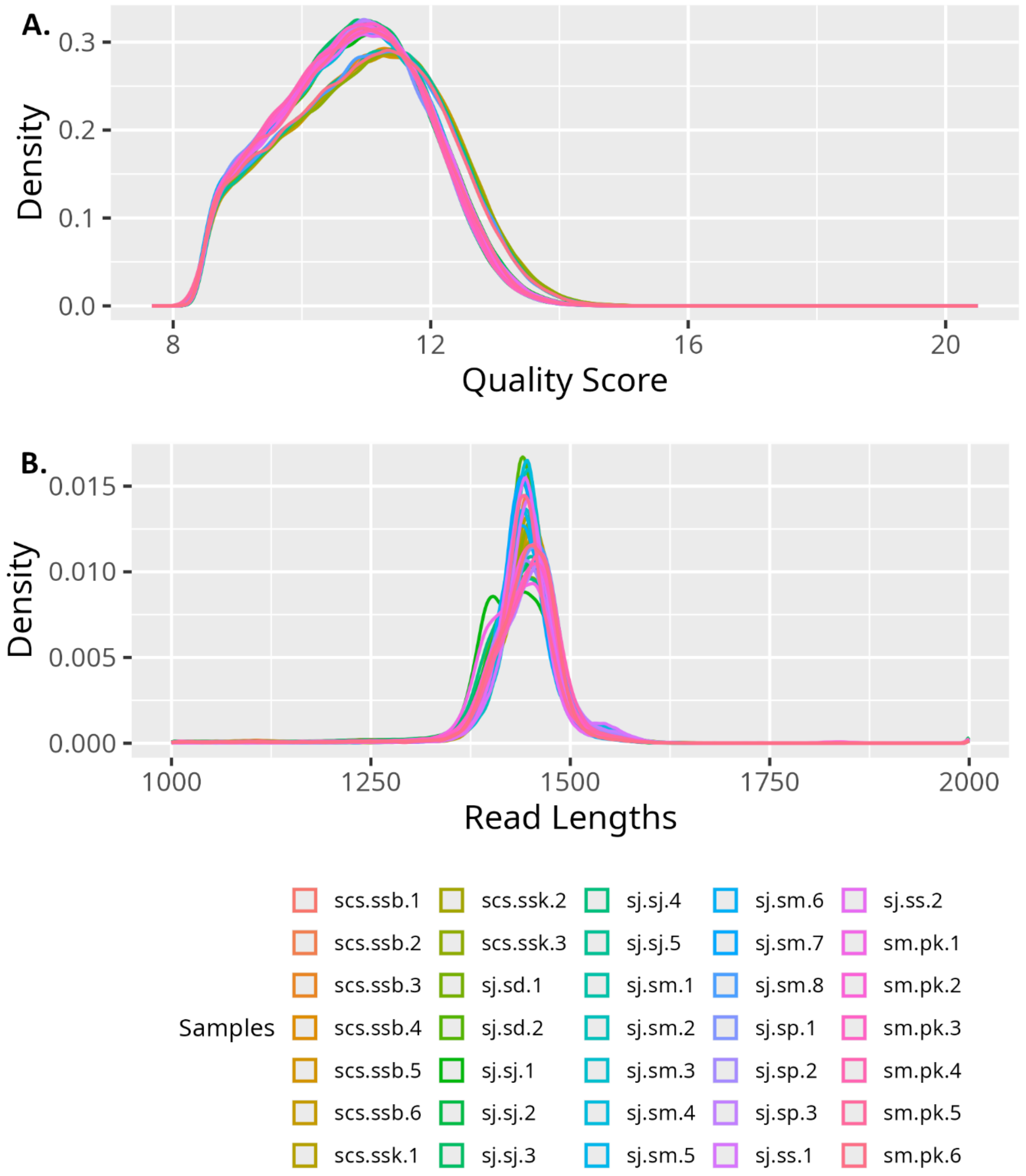

Supplementary Figure 1. Distribution of (A) read quality score and (B) read length across all samples after retaining minimum average read quality score ≥9, minimum sequence length ≥1000 bp and maximum sequence length ≤2000 bp.

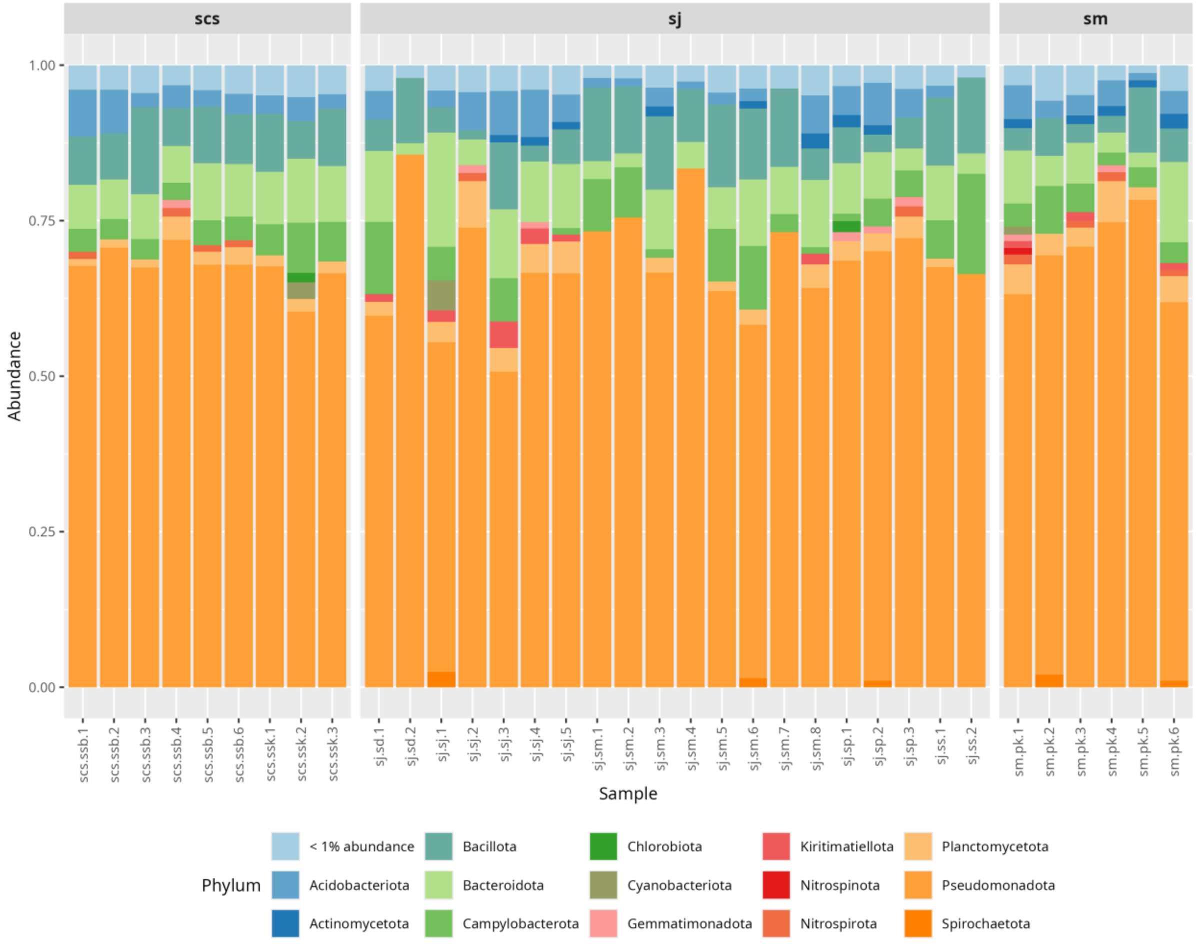

Supplementary Figure 2. Bar plot showing core microbiome structure from each sample. The relative abundance of the core microbiome in each mangrove soil samples of South China Sea (scs), Straits of Johor (sj) and Straits of Malacca (sm) regions.

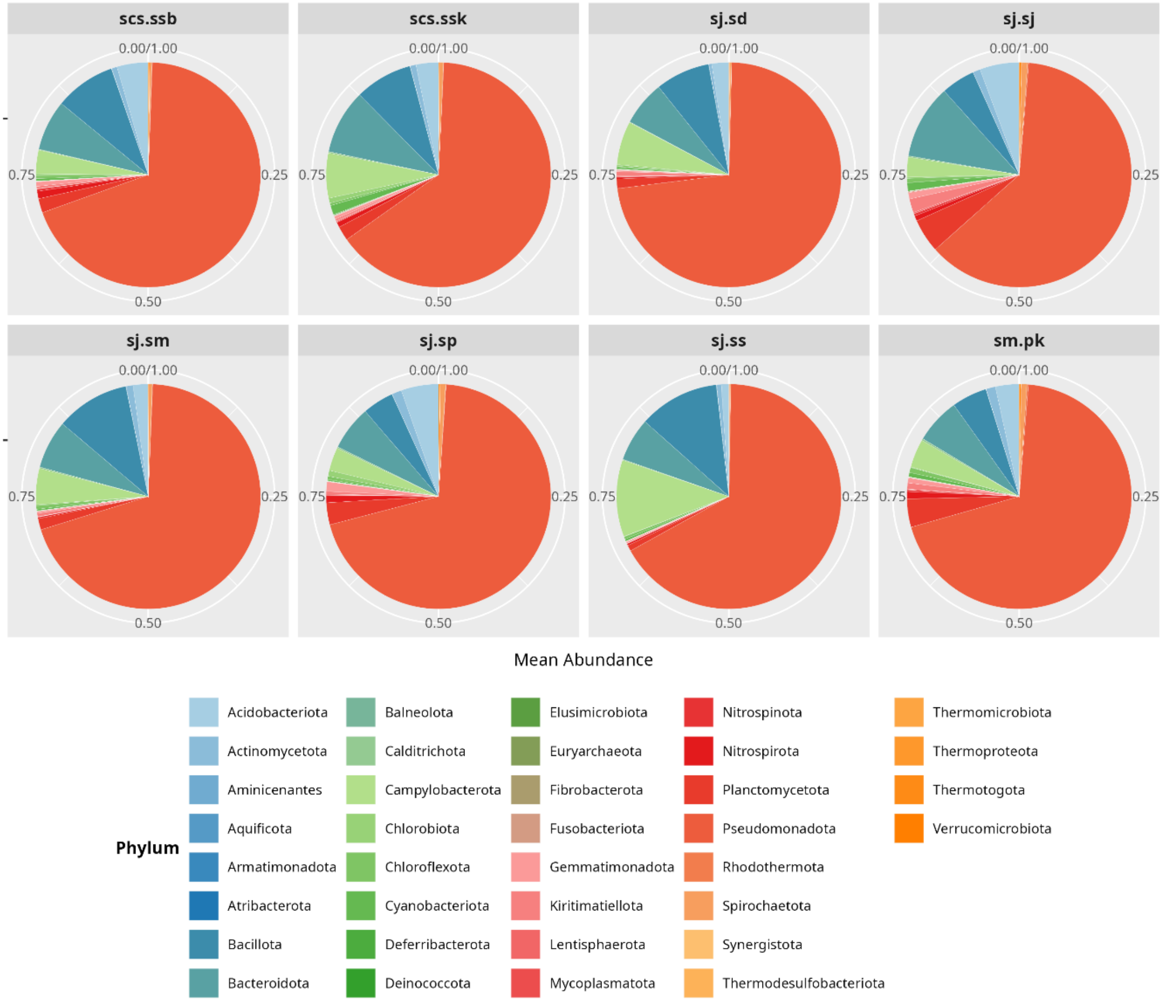

Supplementary Figure 3. Pie chart displaying mean relative abundance of location wise core microbiome. Sungai Sedili Besar (scs.ssb), Sungai Sedili Kecil (scs.ssk), Sungai Danga (sj.sd), Sungai Johor (sj.sj), Sungai Melayu (sj.sm), Sungai Pulai (sj.sp), Sungai Skudai (sj.ss), Pulau Kukup (sm.pk).

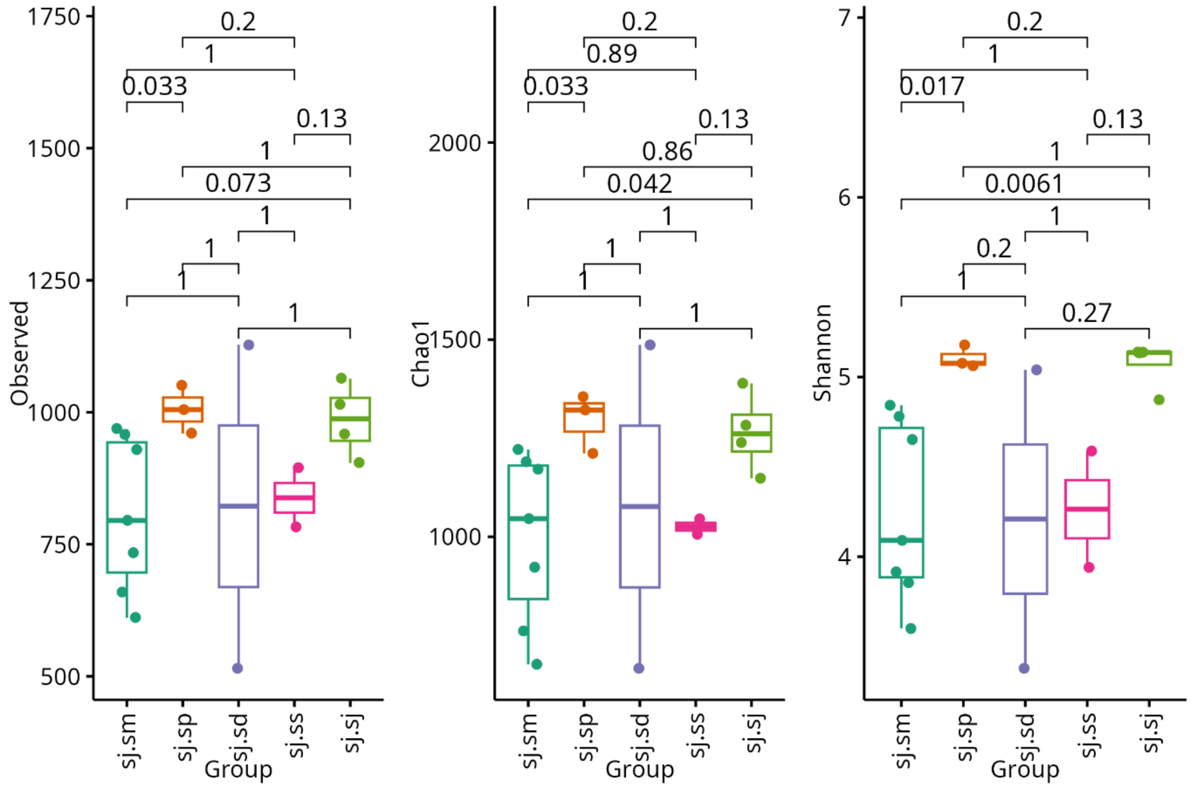

Supplementary Figure 4. Alpha diversity location wise comparison of Straits of Johor (sj). Sungai Melayu (sj.sm), Sungai Pulai (sj.sp), Sungai Danga (sj.sd), Sungai Skudai (sj.ss), Sungai Johor (sj.sj). Statistical significance was defined as $p < 0.05$.

Supplementary Table 1. Characteristics of sampling sites.

Sample Region	Mangrove location	Characteristics	Anthropogenic risks	Sample site code	Coordinates
Straits of Malacca (sm)	1. Pulau Kukup (pk)	Island mangrove within Johor National Parks in the straits of Malacca	Near fishing village, mariculture, and ecotourism, high boat traffic	sm.pk.1, sm.pk.2, sm.pk.3, sm.pk.4, sm.pk.5, sm.pk.6	1°19'36.9"N 103°26'14.2"E, 1°19'30.2"N 103°25'53.5"E, 1°19'35.2"N 103°26'07.7"E, 1°19'39.2"N 103°26'10.2"E, 1°19'26.9"N 103°25'49.9"E, 1°19'49.4"N 103°25'56.0"E
Straits of Johor (sj)	2. Sungai Pulai (sp)	Estuarine mangroves with multiple tributaries joining sungai Pulai	Close to the Port of Tanjung Pelepas (PTP) and industrial area, high boat traffic	sj.sp.1, sj.sp.2, sj.sp.3	1°23'52.4"N 103°32'44.3"E, 1°23'48.0"N 103°32'50.6"E, 1°23'29.0"N 103°33'06.6"E
	3. Sungai Melayu (sm)	Estuarine mangroves with multiple tributaries joining sungai Melayu	Aquaculture, domestic wastewater and urbanisation	sj.sm.1, sj.sm.2, sj.sm.3, sj.sm.4, sj.sm.5, sj.sm.6, sj.sm.7, sj.sm.8	1°27'39.6"N 103°41'08.5"E, 1°27'38.7"N 103°41'06.5"E, 1°27'24.2"N 103°40'56.9"E, 1°28'07.5"N 103°40'46.5"E, 1°27'59.2"N 103°40'06.5"E, 1°27'31.1"N 103°41'57.1"E, 1°27'27.8"N 103°41'43.1"E, 1°27'17.0"N 103°41'32.5"E
	4. Sungai Danga (sd)	Estuarine mangroves with small patch of mangroves on	Near aquaculture ponds, domestic wastewater,	sj.sd.1, sj.sd.2	1°28'10.1"N 103°42'31.2"E, 1°28'36.3"N 103°42'06.5"E

		both sides of sungai Danga	land reclaim and urbanisation		
	5. Sungai Skudai (ss)	Estuarine mangroves with small patch of mangroves on both sides of sungai Skudai	Near aquaculture ponds, domestic wastewater, land reclaim and urbanisation	sj.ss.1, sj.ss.2	1°28'27.8"N 103°43'10.8"E, 1°28'43.9"N 103°42'14.5"E
	6. Sungai Johor (sj)	Estuarine mangroves	Industrial and agricultural runoff, high boat traffic	sj.sj.1, sj.sj.2, sj.sj.3, sj.sj.4, sj.sj.5	1°34'52.0"N 103°58'42.3"E, 1° 34'42.1"N 103°58'56.3"E, 1°34'37.2"N 103°58'55.6"E, 1° 35'21.0"N 103°58'50.1"E, 1° 35'40.2"N 104°00'10.8"E
South China Sea (scs)	7. Sungai Sedili Besar (ssb)	Estuarine mangroves	Agriculture and domestic wastewater	scs.ssb.1, scs.ssb.2, scs.ssb.3, scs.ssb.4, scs.ssb.5, scs.ssb.6	1°56'39.8"N 104°06'09.6"E, 1°56'34.4"N 104°06'13.6"E, 1°56'24.4"N 104°06'14.8"E, 1°56'35.0"N 104°06'20.3"E, 1°56'22.5"N 104°06'25.0"E, 1°56'17.7"N 104°06'25.9"E
	8. Sungai Sedili Kecil (ssk)	Estuarine mangroves	Agriculture and domestic wastewater	scs.ssk.1, scs.ssk.2, scs.ssk.3,	1°50'21.2"N 104°08'55.1"E, 1°50'16.5"N 104°08'47.8"E, 1°50'01.9"N 104°08'58.3"E,

Supplementary Table 2. Surface water quality of sampling sites.

Sample area	Temperature (°C)	pH	DO (mg/L)	Conductivity (mS/cm)
sm.pk	28.82 ± 0.48	6.76 ± 0.45	6.26 ± 0.26	34.8 ± 0.7
sj.sp	30.20 ± 0.40	6.51 ± 0.42	5.83 ± 0.11	30.2 ± 1.2
sj.sm	30.48 ± 0.33	6.27 ± 0.16	7.21 ± 1.76	35.5 ± 0.8
sj.sd	30.80 ± 0.14	6.32 ± 0.16	4.81 ± 0.18	31.5 ± 1.7
sj.ss	30.40 ± 0.14	6.18 ± 0.11	4.77 ± 0.12	31.2 ± 1.1
sj.sj	30.98 ± 0.23	6.23 ± 0.14	6.86 ± 0.75	32.5 ± 0.8
scs.ssk	29.18 ± 0.52	6.91 ± 0.17	7.00 ± 0.13	29.0 ± 0.3
scs.ssb	29.15 ± 0.53	6.92 ± 0.19	7.00 ± 0.22	28.7 ± 0.9

Supplementary Table 3. Expanded source data of the final filtered representative sequence, taxonomical classification and frequency per sample. (See attached EXCEL FILE)
